# Metabolic and Physiological Responses of Trained Working Dogs During the Protection Phase of the “Internationale Gebrauchshunde Prüfungs-Ordnung, Level 1” (IGP1)

**DOI:** 10.3390/vetsci13040355

**Published:** 2026-04-04

**Authors:** Raffaella Cocco, Sara Sechi, Maria Rizzo, Claudia Giannetto, Federica Arrigo, Maria Luisa Pinna Parpaglia, Giuseppe Piccione, Francesca Arfuso

**Affiliations:** 1 Department of Veterinary Sciences, Teaching Veterinary Hospital, University of Sassari, Via Vienna 2, 07100 Sassari, Italy; rafco@uniss.it (R.C.); sarasechilavoro@tiscali.it (S.S.); pinnapar@uniss.it (M.L.P.P.); 2 Department of Veterinary Sciences, University of Messina, Polo University Annunziata, 98168 Messina, Italy; claudia.giannetto1@unime.it (C.G.); federica.arrigo@studenti.unime.it (F.A.); gpiccione@unime.it (G.P.); farfuso@unime.it (F.A.)

**Keywords:** working dogs, energetic metabolism, lactate, muscle enzymes, exercise

## Abstract

This study aimed to evaluate the trends in physiological and metabolic parameters—such as heart rate (HR), respiratory rate (RR), hematological parameters, blood lactate, and serum enzymes (LDH, AST, and CK)—in seven regularly trained working dogs during the protection phase of the IGP1 Working Trial. HR and RR measurements, along with blood samples, were collected at rest (upon arrival at the field), immediately after exercise, and 10 min post-exercise. Statistical analysis revealed significantly higher HR, RR, RBC, hemoglobin (Hb), hematocrit (Hct), and blood lactate values both immediately and 10 min after exercise compared to rest. Creatine kinase (CK) concentrations were significantly higher 10 min post-exercise compared to rest and immediately after exercise. Additionally, a positive correlation was found between blood lactate levels and HR and RR throughout the monitoring period. Preliminarily, these findings suggest that the protection phase of the IGP1 trial involves a mixed energy metabolism, comprising both anaerobic (alactic and lactic) and aerobic pathways. The results also highlight the usefulness of blood lactate and heart rate measurements as practical indicators for assessing fitness and physiological response in working dogs during IGP1 activities.

## Simple Summary

This study evaluated heart rate (HR), respiratory rate (RR), hematological parameters, blood lactate, and serum enzymes (LDH, AST, and CK) in seven trained working dogs during the protection phase of the IGP1 Working Trial. Measurements were taken at rest, immediately after exercise, and 10 min post-exercise. HR, RR, RBC, Hb, Hct, and blood lactate increased significantly after exercise and remained elevated after 10 min. CK levels were significantly higher 10 min post-exercise. Blood lactate showed a positive correlation with HR and RR. Overall, the protection phase appears to involve mixed energy metabolism, including anaerobic (alactic and lactic) and aerobic. Lactate and HR are useful indicators of fitness and physiological response in working dogs.

## 1. Introduction

The central goal of canine sports medicine is the study of scientific methods to overcome the problems inherent in athletic preparation and sporting events in order to obtain better performance from animals. The desired result is to achieve maximum performance with minimum effort [1,2]. The numerous fields of interest that this branch encompasses include genetics, sports physiology and physical training, nutritional science, reproduction and endocrinology, behavioral sciences, orthopedics and traumatology, and doping control [3,4,5]. During physical activity, skeletal muscle requires a continuous production of adenosine triphosphate (ATP) in order to sustain contraction and maintain adequate functional capacity. In exercising dogs, the energy required for muscular work derives from the interaction between aerobic and anaerobic metabolic pathways, whose relative contribution depends mainly on the intensity and duration of the effort [6]. In moderate or prolonged activity, aerobic metabolism predominates and allows efficient ATP production through oxidative phosphorylation, whereas high-intensity exercise promotes a greater activation of anaerobic glycolysis with consequent accumulation of lactate in the muscle and bloodstream [4,6]. Blood lactate concentration is therefore considered a useful indicator of metabolic response to exercise and reflects the balance between lactate production and its clearance during muscular activity. Previous studies conducted on exercising dogs, including agility dogs and other canine athletes, have demonstrated significant variation in lactate levels following physical exertion, supporting its use as a marker of anaerobic metabolism during intense activity [4]. In addition to metabolic changes, physical exercise can induce hematological adaptations aimed at optimizing oxygen delivery to active tissues. Parameters such as red blood cell count (RBC), hemoglobin concentration (Hb), and hematocrit (Hct) are directly related to the oxygen-carrying capacity of the blood and may increase during exercise as a consequence of physiological mechanisms such as splenic contraction, which enhances the availability of circulating erythrocytes and improves oxygen transport to the working muscles [3]. Muscular activity may also influence the circulating concentrations of enzymes associated with muscle metabolism and structural integrity. Among these, creatine kinase (CK), lactate dehydrogenase (LDH) and aspartate aminotransferase (AST) are commonly used as biochemical markers of muscle metabolism and muscular stress induced by physical exercise [5]. In particular, LDH plays an important role in the reversible conversion between pyruvate and lactate, linking anaerobic glycolysis to oxidative metabolism and contributing to the regulation of lactate dynamics during exercise [5]. Regular training is crucial to improve the aerobic capacity of the athlete and to decrease the risk of musculoskeletal damage [7]. In order to evaluate the functional status of muscle tissue, the assessment of the serum concentration of enzymes or proteins known as markers of skeletal muscle activity is of paramount importance. Among these enzymes/proteins, lactate dehydrogenase (LDH), aspartate aminotransferase (AST) and creatine kinase (CK) are suggested as the most useful serum markers of muscle injury [8]. Despite the growing interest by veterinarians and sports physiologists on the metabolic adaptive response of athletic dogs to physical exercise, several studies have already investigated physiological and biochemical changes associated with exercise in different categories of canine athletes, such as agility dogs, sled dogs and racing greyhounds [3,6]. However, limited information is currently available regarding working dogs performing protection exercises, particularly those involved in “Internationale Gebrauchshunde Prüfungs-Ordnung, Level 1” (IGP1), a worldwide dog sport that allows the working potential of a dog, as well as the teamwork of a dog and its handler, to be revealed. In view of the above, the present study aimed to investigate the main physiological and metabolic responses (i.e., hematological parameters, blood lactate, serum LDH, AST, CK, heart and respiratory rate) in regularly trained working dogs involved in IGP1 exercises. The findings may contribute to a better understanding of the physiological and metabolic responses associated with protection exercise in working dogs.

## 2. Materials and Methods

### 2.1. Animal and Experimental Design

**Figure 1:**
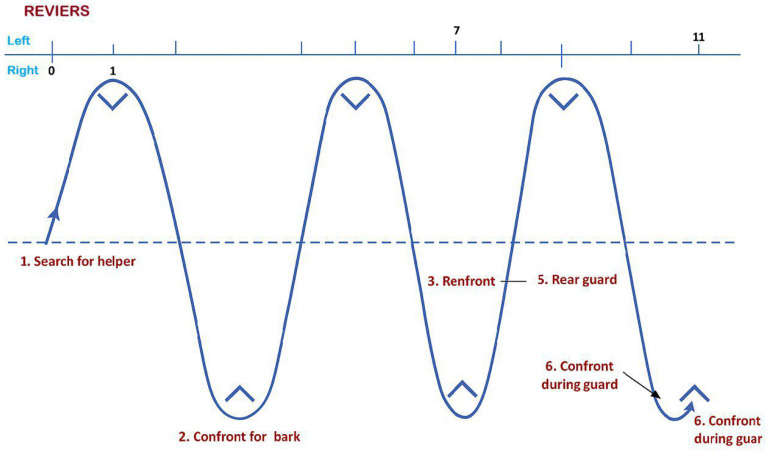
Graphical representation of the physical exercise performed by enrolled working dogs.

The study was carried out according to Directive 2010/63/EU on the protection of animals used for scientific purposes and according to the recommendations of the ARRIVE guidelines. Ethical approval was obtained from the Body Responsible for Animal Welfare and Experimentation of the University of Sassari (OPBSA; Prot. no. 51237, 15 May 2025). This study was conducted on a group of dogs trained for the protection phase of the IGP1 trials at the same training center in Sardinia, Italy, under natural environmental conditions (sunrise 6:30 AM, sunset 8:15 PM, mean temperature 19 °C, mean relative humidity 61%). Seven adult German Shepherd dogs (not neutered and/or spayed; no females in estrus were present during the study), each previously trained in protection work according to IGP standards, were enrolled in the study (Table 1). The protection phase of the IGP1 Working Trial evaluates the dog’s courage, self-confidence, control, and ability to respond to the helper’s actions. It consists of a series of exercises designed to simulate real protection work while assessing stability and obedience under stress. The program includes the following exercises: search for the helper, during which the dog locates the hidden helper in the designated blinds; confrontation and barking, where the dog must alert and hold the helper by barking assertively without biting; prevention of an escape attempt, requiring a decisive and full grip on the sleeve followed by a controlled release; re-attack during the guarding phase, testing the dog’s reactivity and grip stability under renewed provocation; and finally, the long-distance attack, where the dog is sent over a distance to stop a frontal charge by the helper and must again demonstrate a firm, calm grip and precise release on command. Throughout all exercises, the dog is expected to display confidence, determination, and clear control, engaging only the protective sleeve. All tests were conducted on a standardized IGP protection field measuring 100 × 80 m. Six blinds (reviers) were placed alternately, three on the right and three on the left, each spaced 30 m apart and located 30 m from the central axis of the field (Figure 1). This layout conforms to official IGP trial specifications. The mean time required by the dogs to complete the IGP protection course was approximately 4 min and 41 s. The following criteria were selected in order to include dogs in the study: owner’s informed consent for the scientific use of the animal’s data, clinical health confirmed by physical examination, absence of external and internal parasites, and adequate nutritional status and non-aggressive behavior. All enrolled dogs lived in private households with free access to a garden and were fed a high-quality commercial diet once daily (Table 2), adjusted according to body weight and age. Water was available ad libitum. All animals were clinically healthy and free from internal and external parasites. Their health status was evaluated through a clinical exam, including assessment of body condition score, muscle condition score, rectal temperature, heart rate, respiratory rate, appetite, fecal consistency and behavior. All dogs were trained by the same professional instructor and followed the same training program described in Table 3; they were handled by their respective owners during the experimental procedures.

### 2.2. Physiological Parameters Measurement, Blood Sampling and Analysis

From all dogs, the HR and RR were recorded upon their arrival at the field (rest), immediately after exercise (within 3 min from the end of training, 3 min AE), and 10 min from the end of the exercise (10 min AE). Specifically, the HR was taken with a stethoscope while the RR was determined by the observation of the movement of the dog’s flank, and a cycle of one rise and one fall of the flank constituted one breath. At the same time points, from each dog, blood sampling was performed by cephalic vein puncture of the forearm by applying a tourniquet into vacutainer tubes containing EDTA (Terumo Co., Tokyo, Japan) and into whey vacutainer tubes (Terumo Co., Tokyo, Japan). All dogs were habituated to blood collection, and, during both training sessions and the actual blood draws, they received positive reinforcement, such as treats and praise, in order to minimize stress. The blood samples collected into EDTA tubes were immediately tested for blood lactate concentration through a small handheld meter (Accutrend Plus, Roche Diagnostics, Basel/Kaiseraugst, Switzerland), as well as for hematological parameters (i.e., red blood cells, RBC; hemoglobin, Hb; hematocrit, Hct; and white blood cells, WBC). Blood samples collected in whey vacutainer tubes were cooled in an ice-water bath and transported to the laboratory within 2 h of collection, where they were centrifuged at 3000 rpm for 7 min to obtain serum. The obtained sera were used to assess the concentration of LDH, AST and CK through commercially available kits (BioSystems, Barcelona, Spain, catalog numbers B92-11587, B92-11561 and B92-11791, respectively) using an automated analyzer UV spectrophotometer (model Slim SEAC, Florence, Italy).

### 2.3. Statistical Analysis

The Kolmogorov–Smirnov test was used to assess the normal distribution of data. Data passed the normality test (*p* > 0.05) and were analyzed using the one-way analysis of variance (ANOVA) for repeated measures, followed by Tukey’s multiple comparison test for post hoc comparisons. The association between the blood lactate levels and the values of HR, RR, LDH, AST and CK throughout the monitoring period was evaluated using Pearson correlation and linear regression. *p*-values < 0.05 were considered statistically significant. Data analysis was performed using GraphPad Prism v. 9.0.

## 3. Results

**Figure 2:**
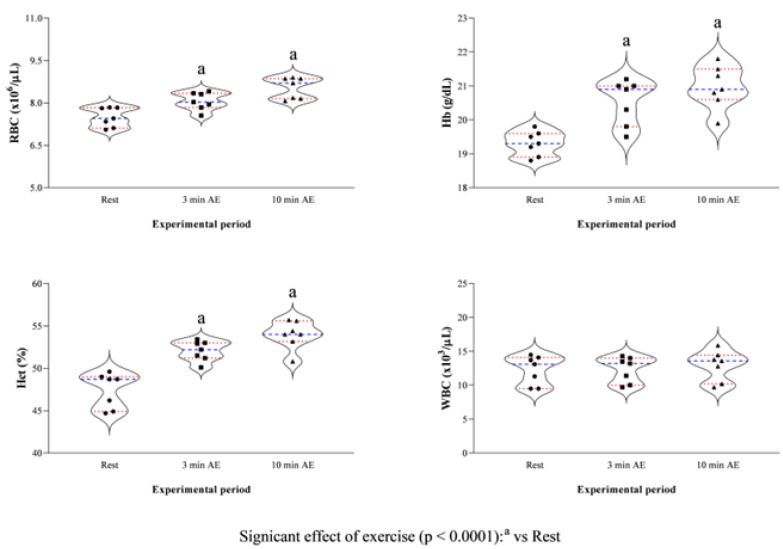
Violin plot showing distributions of the values of hematological parameters (i.e., red blood cells, RBC; hemoglobin, Hb; and hematocrit, HCT) and of white blood cells (WBCs) together with the relative statistical significances recorded upon their arrival at the field (rest), within 3 min after exercise (3 min AE), and 10 min from the end of the exercise (10 min AE). Red dashed lines indicate quartiles; blue dashed lines indicate the mean value; the symbols (i.e., circle, square, and triangle) indicate the values of the respective parameter measured at each time point of the experimental period.

**Figure 3:**
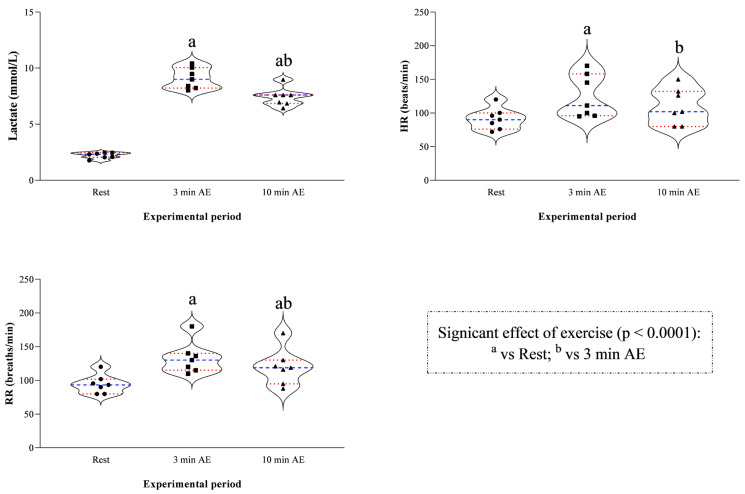
Violin plot showing distributions of the values of blood lactate, heart rate (HR) and respiratory rate (RR) together with the relative statistical significances recorded upon their arrival at the field (rest), within 3 min after exercise (3 min AE) and 10 min from the end of the exercise (10 min AE). Red dashed lines indicate quartiles; blue dashed lines indicate the mean value; the symbols (i.e., circle, square, and triangle) indicate the values of the respective parameter measured at each time point of the experimental period.

**Figure 4:**
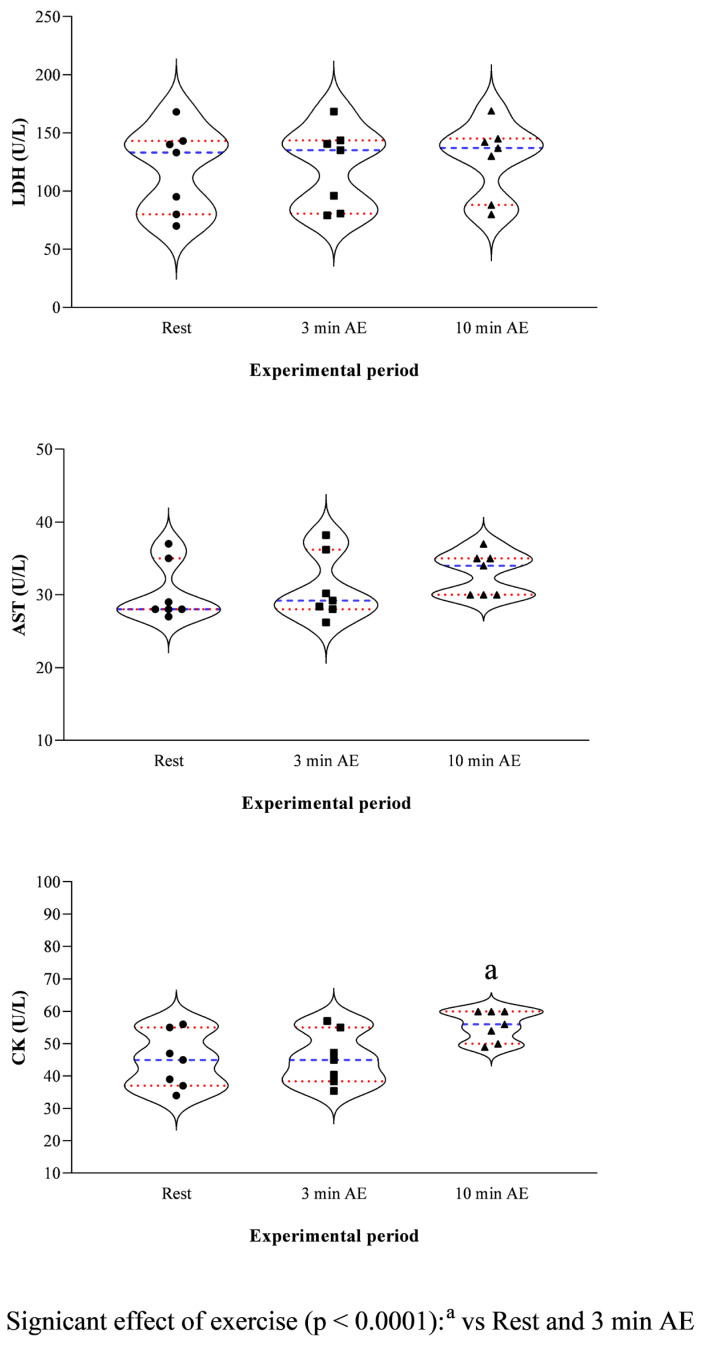
Violin plot showing distributions of the values of serum lactate dehydrogenase (LDH), aspartate aminotransferase (AST) and creatine kinase (CK) together with the relative statistical significances recorded upon their arrival at the field (rest), within 3 min after exercise (3 min AE) and 10 min from the end of the exercise (10 min AE). Red dashed lines indicate quartiles; blue dashed lines indicate the mean value; the symbols (i.e., circle, square, and triangle) indicate the values of the respective parameter measured at each time point of the experimental period.

**Figure 5:**
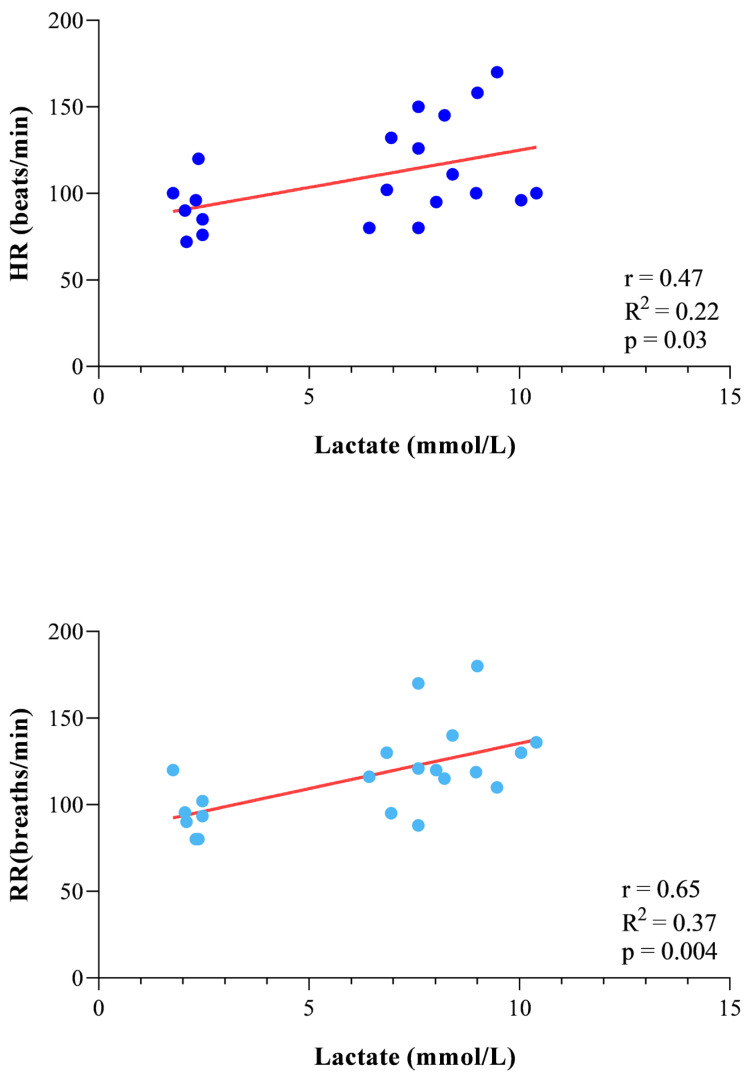
Relationship between the concentration of blood lactate and the values of heart rate (HR) and respiratory rate (RR) measured in the working dogs throughout the experimental period.

**Figure 6:**
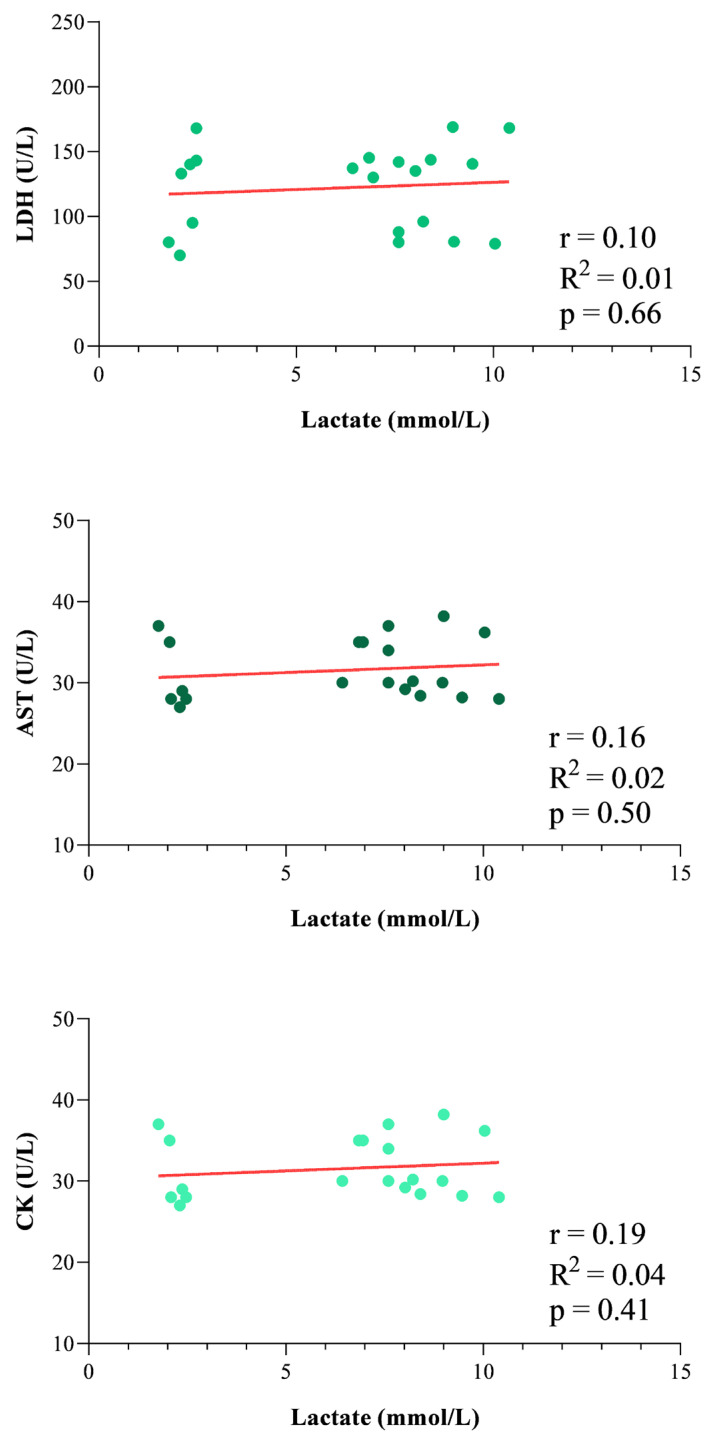
Relationship between the concentration of blood lactate and the values of serum lactate dehydrogenase (LDH), aspartate aminotransferase (AST) and creatine kinase (CK) measured in the working dogs throughout the experimental period.

The study’s results are expressed as mean ± standard deviation (±SD). According to the statistical analysis results, erythrocyte indices showed higher values at 3 min AE and 10 min AE compared to rest (RBC F_(2)_ = 20.94, *p* < 0.0001, Hb F_(2)_ = 27.13, *p* < 0.0001, and Hct F_(2)_ = 29.31, *p* < 0.0001, Figure 2), whereas the WBC levels showed no statistically significant difference throughout the experimental period (F_(2)_ = 2.89, *p* = 0.13, Figure 2). The concentration of blood lactate, as well as the values of HR and RR, was higher at 3 min AE and 10 min AE compared to rest (F_(2)_ = 11.14, *p* < 0.0001, Figure 3). The LDH and AST concentrations showed no significant changes throughout the experimental period (LDH F_(2)_ = 2.73, *p* = 0.15; AST F_(2)_ = 3.62, *p* = 0.10, Figure 4); in contrast, the values of CK increased 10 min after the end of exercise compared to the values measured at rest and 3 min after exercise (F_(2)_ = 13.70, *p* < 0.0001, Figure 4). A significant positive correlation between the concentrations of blood lactate and the values of HR and RR was observed throughout the experimental period (Figure 5), whereas no significant correlation was found between the concentration of lactate and the values of LDH, AST and CK (Figure 6).

## 4. Discussion

In sport veterinary medicine, scant data are available on muscle biomarkers and physiological parameters of trained dogs following exercise; specifically, few studies have investigated the variations of these biomarkers during exercise in racing greyhounds [9], sled dogs [10,11], Beagle dogs performing treadmill exercise [5,6,12], and agility dogs [2]. The results gathered in these previous studies suggest that, in dogs, protracted endurance workouts and high-intensity racing made mild but significant elevations in the skeletal muscle biomarkers, as well as biochemical and hematological changes. To date, little is known about how the type of exercise faced by working dogs affects their performance. To the best of the authors’ knowledge, the present study, although preliminary, provides new data on this field. Specifically, according to the results, the erythrocyte indices, blood lactate, heart and respiratory rates showed significant changes in working dogs performing physical activity within the protection phase of the IGP trials. Regularly trained working dogs included in this study showed significant physiological, cardiovascular and respiratory responses to exercise, as indicated by the changes observed in blood lactate levels and erythrocyte indices, as well as the heart rate and respiratory rate. The lactate values obtained from the dogs investigated in the current study by means of Accutrend Plus (Roche Diagnostics, Switzerland) were consistent with findings from other commercially available devices [13,14,15,16,17]. This indicates good agreement with laboratory analyses. Furthermore, previous validation studies have shown that portable lactate analyzers, such as the Accutrend system, exhibit a standard error of estimate (SEE) of approximately 0.33 mmol/L when compared to laboratory reference instruments, indicating a relatively small measurement error [17]. Although this level of error should be considered, lactate values must be interpreted by taking into consideration the fact that all measurements were performed using the same device; therefore, any minor measurement errors would have been distributed across all sampling points, confirming greater methodological consistency in the study. Regarding hematology, the higher RBC, Hb and Hct values observed in dogs following exercise compared to the rest condition are consistent with splenic contraction as previously found in athletic dogs [2,4,12]. In contrast to what was previously found in exercising dogs [18], no exercise-induced changes in WBC values were observed in working dogs. The lack of change in WBC count in dogs following exercise observed in the current study agrees with other investigations carried out on exercising dogs [12,19]. An increased WBC count following exercise is likely to happen secondary to mobilization of the marginal pool of segmented neutrophils and with the release of lymphocytes from the spleen [20]. Moreover, it has been recognized that athletes are at increased risk of developing inflammation [21]. The clinical check of the working dogs enrolled in the current study did not highlight any inflammatory processes associated with the musculoskeletal system, including joints, tendons, ligaments, or periosteum. The trend of blood lactate values observed in working dogs following exercise suggests a utilization of anaerobic metabolism in contracting muscles [4]. The normal reference range for lactate for healthy dogs has been reported as 0.3 mmol/L to 2.5 mmol/L [22]; therefore, the blood lactate levels measured in all investigated working dogs (2.22 ± 0.25 mmol/L) fall within the normal range at rest condition, whereas the lactate levels recorded 3 and 10 min after the end of exercise exceed the metabolic or anaerobic lactate threshold of 4.0 mmol/L suggested for exercising dogs, including working ones [23]. The mean values of blood lactate levels recorded in working dogs 3 min (7.43 mmol/L) and 10 min (9.08 mmol/L) after exercise were higher than those found in agility dogs after submaximal exercise (4.56 mmol/L) [22], in foxhounds after maximal exercise (2.71 mmol/L) [24], and in Labrador retrievers after field trial competitions (2.18–3.15 mmol/L) [25]. These differences in lactate levels in dogs enrolled in physical activity are probably related to the longer duration of exercise in foxhounds, Labrador retrievers and agility dogs and, consequently, to the greater involvement of aerobic paths, as has been previously confirmed in horses and dogs [22,26]. In contrast, the lactate levels in working dogs were markedly lower than the values reported for racing greyhounds (29 mmol/L) [27,28] performing maximal exercise. On the one hand, the results suggest an anaerobic metabolism; on the other hand, although an increasing trend of HR was observed after exercise in working dogs, the maximum HR recorded in the studied dogs was 170 beats/minute, a value well below the maximum heart rates for dogs reached during effort (300 beats/min) [22]. However, as the heart rate deflection point was not specifically evaluated in the present study, it cannot be determined whether the dogs exercised above this physiological threshold. This data, together with a hint of a decrease in lactate values 10 min after the end of the exercise, seems to suggest proper adaptation of the animals to the trial to which they were subjected. As expected, the RR values followed the same trend as the HR values, suggesting enhanced oxygen requirements [12,29,30]. An increase in the oxygen demand linked to the work done and the requirement of a better alveolar ventilation to remove the produced carbon dioxide could justify the significant rise in RR values in exercising dogs [31]. According to results gathered in the study, it could be stated that the working dogs used a mixed metabolism pathway, comprising both anaerobic (lactic) and aerobic metabolism. According to the protection phase of the IGP1 Working Trial, the dogs used aerobic metabolism during the search phase and anaerobic lactic metabolism during the attack phase. As a matter of fact, before carrying out exercise, the dogs were trained regularly, and the training program specifically included having the dogs carry out exercises that activated the two metabolic pathways (anaerobic alactatic and aerobic). This training approach is consistent with conditioning programs commonly adopted in working trial dogs, where both aerobic and anaerobic metabolic pathways are stimulated to support the physiological demands of protection work. Notably, a strong positive correlation was found between the concentration of blood lactate and the values of HR and RR recorded in working dogs throughout the experimental period. This observation does not seem surprising because HR, as well as RR, are linked with energy spending [32]. The relationship is clear: the greater the energy expenditure, the greater the need for oxygen in the muscles and, at the same time, the more carbon dioxide and hydrogen ions to eliminate, thus increasing the HR, RR, and lactate accumulation. These findings suggest that HR and RR increase in parallel with metabolic demand during exercise and may therefore represent useful indicators of the physiological response to exercise under field conditions [33,34,35]. Regarding the enzymatic activity of muscle herein investigated by the assessment of the LDH, AST and CK concentrations, only the CK showed a significant increase 10 min after exercise, while LDH and AST showed no significant differences attributable to an exercise effect. Similar trends in muscle biomarkers following exercise have been previously reported in dogs [5,11]. In the current study, the post-exercise sampling schedule was limited to three time points due to ethical constraints related to animal welfare, as the dogs were privately owned. Therefore, sampling was performed up to 10 min post-exercise to evaluate the immediate physiological response under field conditions. It should be noted that LDH and AST concentrations may reach their peak several hours after intense exercise, generally between 6 and 12 h after the exercise. Therefore, the sampling times adopted in the present study may not have captured the maximal post-exercise response of these enzymes. Moreover, the blood lactate levels were not found to be correlated with those of LDH, AST and CK in working dogs throughout the experimental period. Of note, the values of these muscle parameters fell within the reference ranges indicated for the canine species [36] throughout the experimental study, suggesting that, despite the high blood lactate levels found in dogs after exercise, the muscle was not in a suffering state. It is known that blood lactate concentration denotes the glycolytic activity in skeletal muscle, and it is related to the intensity of muscular exertion [18]. For a long time, lactate was seen as the archenemy of athletic performance, as its levels were associated with muscle fatigue following exercise, declining muscle force or power output, and, thus, limitation in performance. This statement is now considered too simplistic. In recent decades, scientific thought has changed, with new insights into the role of lactate in energy metabolism shifting this original perspective [37]. It has been proposed that lactate is not restricted to anaerobic conditions; it also plays a crucial role in cell signaling during exercise and represents a key energy substrate that can be promptly used by many tissues [37].

Some limitations of the present study should be acknowledged. The small sample size and the absence of a control group may limit the statistical power and the generalization of the results. Moreover, the physiological response observed was evaluated only during a specific phase of the IGP1 trial. Therefore, further studies involving a larger number of working dogs and different types of working activities are needed to better characterize the metabolic response associated with protection exercises.

## 5. Conclusions

Dogs used in sports and/or working roles need to reach an adequate level of physical preparation in order to meet the physiological demands of their activities. The results of this study, although preliminary, highlight that the energy pathways used in the defense section of IGP1 trials are mixed, comprising both anaerobic (alactic and lactic) and aerobic metabolism. The observed variations in blood lactate concentration and cardiorespiratory parameters indicate measurable physiological responses in working dogs during the protection phase of IGP1 trials. Given the limited data currently available on the adaptive energetic responses to exercise in working dogs, further investigations are warranted to better characterize the physiological demands associated with protection work. In this regard, the results gathered in this preliminary study could represent a starting point for investigations into high-performance sporting dogs and the establishment of protocols for health preservation and fitness maintenance.

## Figures and Tables

**Table 1:** Signalment data for enrolled working dogs.

Breed	N	Age	Gender	N	Body Weight
German Shepherd	7	Median 2.5 years	Males	5	Mean 34.9 ± 3.2 kg
		Median 4 years	Females	2	Range 34.0 ± 1.0

**Table 2:** Nutritional formulation of the high-energy, highly digestible commercial diet administered to the enrolled dogs.

Nutrient	Composition (% DM)
Crude Protein	28–32%
Crude Fat	18–22%
Crude Fiber	2.0–3.0%
Crude Ash	6–8%
Moisture	≤10%
Calcium	1.2–1.5%
Phosphorus	0.9–1.1%
Omega-6	3.0–4.0%
Omega-3	0.5–0.8%
ω6/ω3 Ratio	5:1–7:1
L-Carnitine	200–400 mg/kg

**Table 3:** Training program adopted for each working dog before the study.

Weeks	Training Type	Exercise Details	Frequency (Days)	Rest Type and Duration	Additional Training
I	Jump training	1 set of 5 jumps	Mon, Wed, Fri	—	3 km run at trot (8 km/h)
II	Jump training	2 sets of 5 jumps	Mon, Wed, Fri	2–3 min passive rest	4 km run at trot (8 km/h)
III	Jump training	3 sets of 5 jumps	Mon, Wed, Fri	2 min passive rest	5 km run at trot (8 km/h)
IV	Jump training	3 sets of 5 jumps	Mon, Wed, Fri	2 min passive rest	6 km run trot (at 8 km/h)
V	Short-distance speed training	2–300 m runs	Mon, Wed, Fri	3 min active + 1 min passive (massage)	Weighted vest (3 kg), 10 min run at trot (6 km/h)
VI	Short-distance speed training	2–300 m runs	Mon, Wed, Fri	3 min active + 1 min passive (massage)	Weighted vest (3 kg), 10 min run at trot (6 km/h)
VII	Short-distance speed training	2–300 m runs	Mon, Wed, Fri	3 min active + 1 min passive (massage)	Weighted vest (3 kg), 10 min run at trot (6 km/h)
VIII	Short-distance speed training	2–300 m runs	Mon, Wed, Fri	3 min active + 1 min passive (massage)	Weighted vest (3 kg), 10 min run at trot (6 km/h)
IX	Combined training	1 set of 5 jumps + short runs (2–300 m)	Mon, Wed, Fri	3 min active + 1 min passive (massage)	Weighted vest (4 kg), 10 min run at trot (6 km/h)
X	Combined training	1 set of 5 jumps + short runs (2–300 m)	Mon, Wed, Fri	3 min active + 1 min passive (massage)	Weighted vest (4 kg), 10 min run at trot (6 km/h)
XI	Combined training	1 set of 5 jumps + short runs (2–300 m)	Mon, Wed, Fri	3 min active + 1 min passive (massage)	Weighted vest (5 kg), 10 min run at trot (6 km/h)
XII	Combined training	1 set of 5 jumps + short runs (2–300 m)	Mon, Wed, Fri	3 min active + 1 min passive (massage)	Weighted vest (5 kg), 10 min run at trot (6 km/h)

## Data Availability

The original contributions presented in this study are included in the article. Further inquiries can be directed to the corresponding author.
